# Identification of the human cerebral cortical hemodynamic response to passive whole-body movements using near-infrared spectroscopy

**DOI:** 10.3389/fneur.2023.1280015

**Published:** 2023-12-13

**Authors:** Yue Zhao, Yue Wei, Yixuan Wang, Richard H. Y. So, Chetwyn C. H. Chan, Raymond T. F. Cheung, Arnold Wilkins

**Affiliations:** ^1^HKUST-Shenzhen Research Institute, Shenzhen, China; ^2^Department of Industrial Engineering and Decision Analytics, Hong Kong University of Science and Technology, Kowloon, Hong Kong SAR, China; ^3^Department of Basic Psychology, School of Psychology, Shenzhen University, Shenzhen, China; ^4^Bio-Engineering Graduate Program, School of Engineering, Hong Kong University of Science and Technology, Kowloon, Hong Kong SAR, China; ^5^Department of Psychology, The Education University of Hong Kong, Tai Po, Hong Kong SAR, China; ^6^Department of Medicine, School of Clinical Medicine, University of Hong Kong, Pokfulam, Hong Kong SAR, China; ^7^Centre for Brain Studies, University of Essex, Colchester, United Kingdom

**Keywords:** hemodynamics, whole-body motion, near-infrared spectroscopy (NIRS), vestibular, cerebral cortical response

## Abstract

The human vestibular system is crucial for motion perception, balance control, and various higher cognitive functions. Exploring how the cerebral cortex responds to vestibular signals is not only valuable for a better understanding of how the vestibular system participates in cognitive and motor functions but also clinically significant in diagnosing central vestibular disorders. Near-infrared spectroscopy (NIRS) provides a portable and non-invasive brain imaging technology to monitor cortical hemodynamics under physical motion.

**Objective:**

This study aimed to investigate the cerebral cortical response to naturalistic vestibular stimulation induced by real physical motion and to validate the vestibular cerebral cortex previously identified using alternative vestibular stimulation.

**Approach:**

Functional NIRS data were collected from 12 right-handed subjects when they were sitting in a motion platform that generated three types of whole-body passive translational motion (circular, lateral, and fore-and-aft).

**Main results:**

The study found that different cortical regions were activated by the three types of motion. The cortical response was more widespread under circular motion in two dimensions compared to lateral and fore-and-aft motions in one dimensions. Overall, the identified regions were consistent with the cortical areas found to be activated in previous brain imaging studies.

**Significance:**

The results provide new evidence of brain selectivity to different types of motion and validate previous findings on the vestibular cerebral cortex.

## 1 Introduction

It has long been recognized that the cerebral representation of vestibular signals plays an important role in motion perception (1, 2), posture, and oculomotor function (3–5). Moreover, recent studies have demonstrated that the vestibular signals contribute to many higher cognitive functions, such as spatial cognition and memory (6, 7), self-consciousness (8), and body representations (9). Clinical studies have shown that patients with vestibular disorders suffer from a range of cognitive impairments (10). Exploring how the cerebral cortex responds to vestibular signals is not only valuable for a better understanding of how the vestibular system participates in cognitive and motor functions (5, 6, 11) but also clinically significant in diagnosing central vestibular disorders (12). However, our current understanding of the vestibular information processing in the human cerebral cortex is still limited (11). Due to the limitations of most of the neuroimaging techniques that have, to date, been mostly used to explore the human vestibular cortex, the cerebral cortical responses of humans to real physical motion stimuli are still poorly documented.

Our current knowledge of the vestibular regions in the human cerebral cortex mainly comes from two neuroimaging techniques: functional magnetic resonance imaging (fMRI) and positron emission tomography (PET) (3, 13, 14). Previous studies have identified a large vestibular network outside the brainstem that is distributed extensively throughout the brain, encompassing various regions such as the insula area, the temporo-parietal junction (TPJ; including the superior temporal lobe, inferior parietal lobe, and temporo-parietal region), the lateral occipital cortex (LOC), the precentral and postcentral gyri, the precentral sulcus, the dorsolateral prefrontal cortex, the thalamus, and the cerebellum (15–19). Due to the prohibition of head movements inside the imaging scanner, these methods (fMRI and PET) are unable to capture cortical responses to real physical motion stimuli and must use alternative stimuli to activate vestibular responses. These alternative stimuli are mainly caloric vestibular stimulation (CVS), galvanic vestibular stimulation (GVS), neck vibration, and auditory stimulation (15, 17–20). For example, in caloric stimulation, cold/warm water or air is injected into the ear canals of the subjects by means of which the vestibular receptors or nerves are considered to be activated (16, 21). Apart from the use of alternative stimulation in neuroimaging scanners, a few studies have applied direct intracranial stimulation to identify the specific vestibular cortex (22). For example, a lateral cortical temporoparietal area has been identified as the temporo-peri-Sylvian vestibular cortex (TPSVC) using the intracranial electric stimulation of 260 patients with partial epilepsy (23). The results indicated that rotatory sensation can be elicited by electric stimulation directly applied to the TPSVC. Intracranial stimulation on the parietal operculum and the superior and middle temporal gyri has also been shown to elicit sensations of pitch and yaw rotations.

However, the use of alternative stimulation has several limitations that cannot be overlooked. First, unlike the stimulation from or by actual physical movement, alternative stimulation often includes additional interfering inputs unrelated to the motion signal. For example, auditory stimulation can elicit auditory sensations that are unrelated to motion perception, while the CVS can evoke sensations of heat. Hence, the activated cortical regions may not be entirely devoted to motion perception functions. Furthermore, applying alternative vestibular stimulation in a neuroimaging scanner may induce sensory conflicts between vestibular signals (which indicate self-motion) and signals from other motion perception sensors (e.g., visual, auditory, somatosensory, and interoceptive system), which indicate that the participant is stationary in the scanner. Cortical regions, such as the temporo-parietal region, activated under such conditions may be involved in monitoring, processing, and resolving these sensory conflicts (24, 25) rather than vestibular functions. Although direct intracranial stimulation may not have these concerns, it cannot be applied to general healthy participants. Finally, alternative stimulation methods may pose challenges in simulating specific movement directions due to the simultaneous activation of multiple vestibular elements (11). For instance, CVS can activate the horizontal, anterior, and posterior semicircular canals simultaneously. Moreover, GVS can activate multiple afferent fibers from receptors that typically do not activate together during physical head movements. This effect restricted the exploration of cerebral cortical responses to poorly specified motion signals, thereby hindering a comprehensive understanding of vestibular functions (11, 26).

Some portable technologies have the potential to overcome these limitations. Two commonly used portable non-invasive technologies in neuroscience are electroencephalography (EEG) and functional near-infrared spectroscopy (fNIRS). Each technology has its own strengths, advantages, and limitations. EEG measures brain electrical activity with excellent temporal resolution (27). On the other hand, fNIRS detects hemodynamic changes in the cerebral cortical regions (28, 29) and offers good resistance to motion artifacts (30, 31), as well as better spatial resolution compared to EEG (27). Currently, there is a scarcity of research using fNIRS in this context. In this study, fNIRS was chosen primarily for its portability and ability to measure hemodynamic signals, facilitating comparison with existing fMRI/PET data obtained using alternative stimulation methods. Additionally, fNIRS demonstrated good resistance to motion artifacts, making it suitable for capturing cerebral cortical responses during real physical motion stimuli.

**Figure 1:**
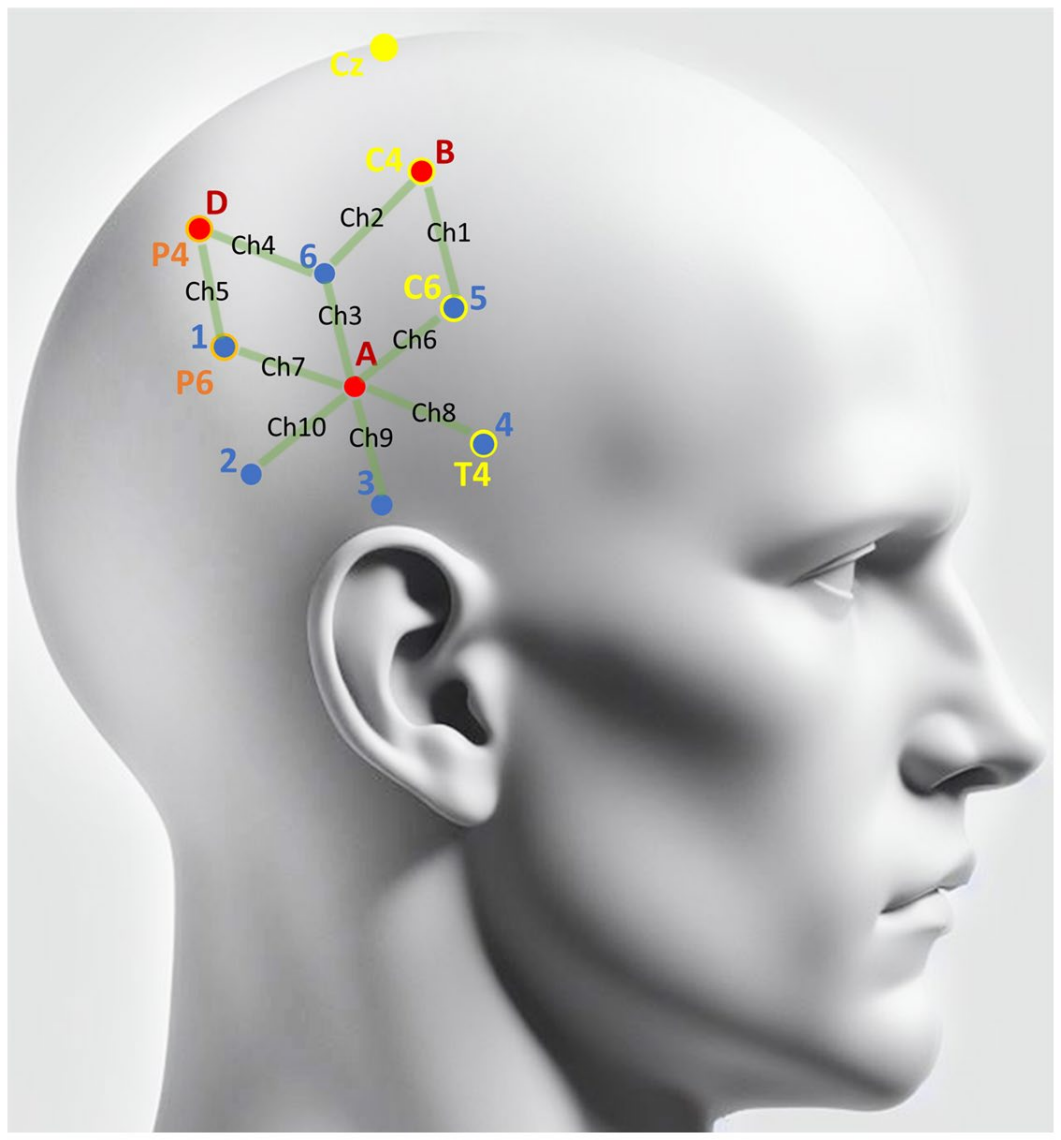
Scalp locations of detectors and light sources. Red dots denote the spatial coordinates of the three NIRS detectors (A\B\D). Blue dots indicate the locations of the six NIRS light sources (1–6) that were applied to the right hemisphere scalp to illuminate the brain tissue with near-infrared light. Green lines denote the 10 channels covered by NIRS. Yellow markers are the landmarks from the 10–20 system utilized to locate the detectors and light emitters. As illustrated, the light source 4 was overlapped with T4, light source 5 overlapped with C6, and the detector B overlapped with T4. Orange markers are the landmarks determined based on yellow markers. As illustrated, the light source 1 overlapped with P6, and detector D overlapped with P4.

Instead of using alternative stimulation to activate the vestibular cerebral cortex, this study used real physical motion generated by a motion platform (Figure 1A) in different directions on the horizontal plane. NIRS was installed in the motion platform (Figure 1B) to investigate cortical regions that respond to actual physical motion and to identify distinct cortical areas that are activated by different types of passive translational motion conditions. In this study, the level of oxygenated hemoglobin (HbO) in the targeted cortical areas, as measured by fNIRS, was considered as the dependent variable to reflect brain activity. The areas of activation by whole-body passive motion should be more relevant to motion processing than the resolution of sensory conflict by virtue of the congruency between signals from vestibular and other sensory afferents. We hypothesized that (1) the HbO level detected in the cortical region related to vestibular functions should significantly increase under the presence of passive motion and (2) the HbO level detected in these vestibular regions should differ in response to the three different types of motion (circular, lateral, and fore-and-aft).

## 2 Methods

### 2.1 Apparatus and NIRS probe placements

In this study, a custom-designed motion platform was used to generate physical motion stimuli. The platform, constructed at the Hong Kong University of Science and Technology (HKUST), consisted of a 4 m × 3 m fully enclosed test platform supported by precision machined rails and custom-built sliding bearings. It was capable of movement along the fore-and-aft axis (x) and lateral axis (y). The motion was enabled by an electromagnetic actuation mechanism (see Appendix I.5 for more details).

An NIRS system was installed on the motion platform. The NIRS system (Imagent ISS Inc., Champaign IL) has 16 light sources and 4 detectors. The light sources used were laser diodes to generate discrete wavelengths of 690 nm and 830 nm with a mean power of 1 mW. Each 690 nm light source and each 830 nm light source were paired up, and thus, a combined light source was used to shine light into the targeted location. Standard SMA905 connectors and 1.0 mm core diameter fibers were used to transmit the light from the sources to the scalp of a subject. The light was modulated at a frequency of 110 MHz. Data were sampled at 12.5 Hz.

The light fiber probes and detectors were located in accordance with the 10–20 system (32). The Cz, C4, and T4 sites of the 10–20 system were chosen as landmarks in positioning the light sources and detectors on the right hemisphere. The line connecting Pz and Oz was the landmark for positioning the detector on the occipital lobe. Light source 4 was placed exactly at T4, and detector B was placed exactly at C4. After the relative locations for light source 4 and detector B were fixed, the locations of other detectors and light emitters were determined relative to them (see Figure 1 for illustration).

The optical fibers were attached to a flexible, but not elastic, rubber cap. The cap was snugly fastened around each subject’s head with a nylon tape. Hairs between the optical fibers and the scalp were carefully moved out of the way before data collection. The positions of the optical fibers, detectors, and light sources were further secured by winding a black bandage along the cap.

In this study, each pair of detectors and light source with the distance between them was denoted as a channel. Three detectors and six light sources, forming a total of 10 channels, were placed on the targeted brain areas of the right hemisphere to measure the signal responding to physical motion (see Figure 1 for light source and detector locations). The placement of these NIRS channels was primarily based on the common regions reported in previous MRI and PET studies using various alternative vestibular stimuli (3, 33). We focused on the right hemisphere as most studies showed a right hemispheric dominance of vestibular cortical response in right-handed subjects (34, 35). To provide reference control, one additional detector and two more light sources were placed on the scalp above the occipital lobe, which formed two additional channels. One of the two channels recorded signals from the visual area, while the light emitter of the other channel was deliberately kept away from the scalp to ensure that the detector could collect signals with no nearby infrared light source. These two channels were both labeled as blank and utilized to control environmental noise.

### 2.2 Participants

Twelve right-handed male subjects (23–28 years old, mean 24.5) participated in this study after giving informed consent. None of them had any history of neurological or psychiatric disease. All participants were free of any vestibular injury or medical treatment. Alcohol and caffeine beverages were forbidden for 10 h before the experiment for all participants. This study was approved by the human subject and ethics committee of the HKUST.

### 2.3 Testing protocols

#### 2.3.1 Motion stimuli

**Figure 2:**
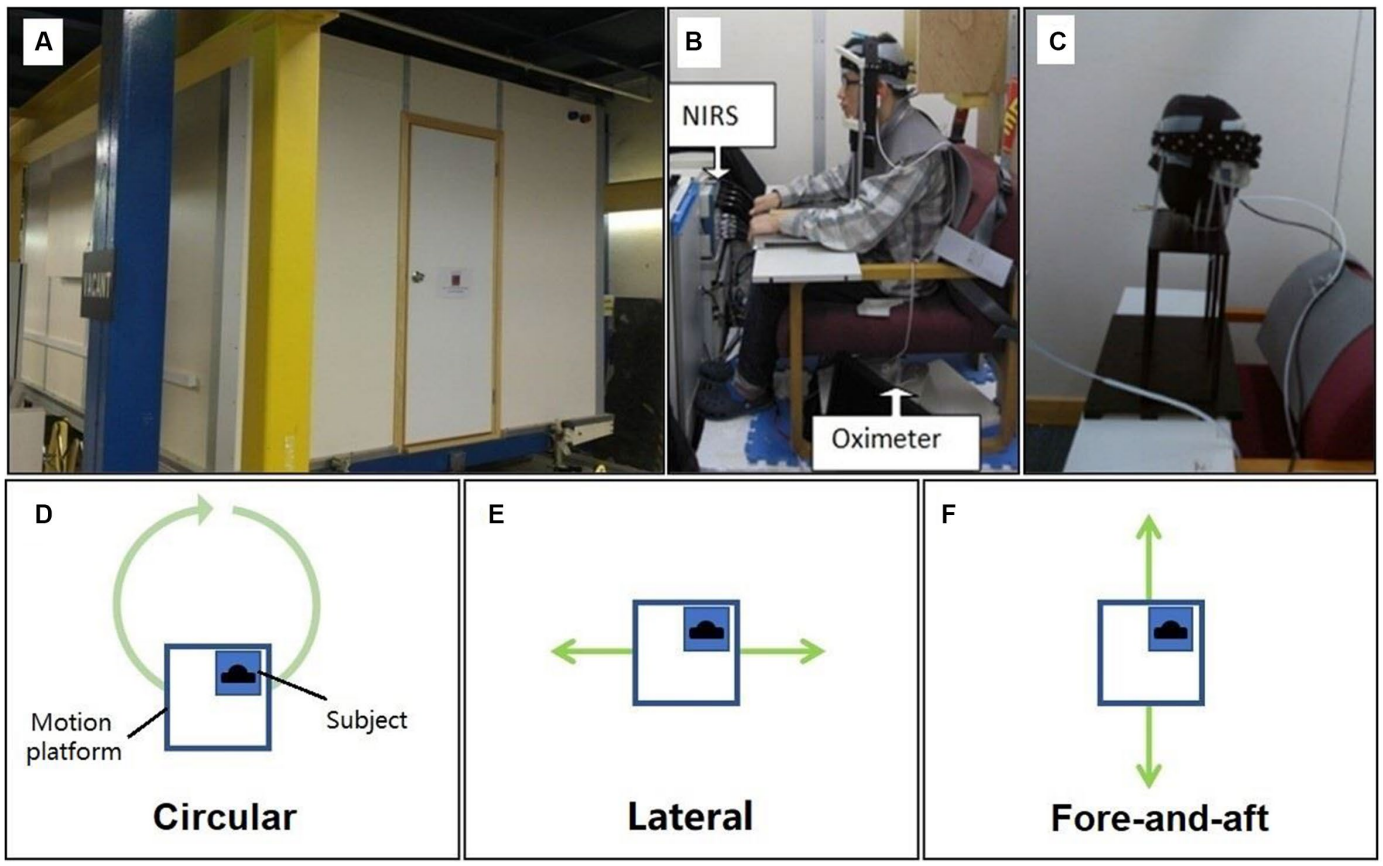
Motion platform and three types of motion stimuli. **(A)** The outside photo of the motion platform. **(B)** The photo of the NIRS and the oximeter setup inside the motion platform. **(C)** The photo of plastic manikin head in validation tests. **(D)** The top view of motion platform and the illustration of circular motion stimuli applied in the study. **(E)** The illustration of lateral motion stimuli. **(F)** The illustration of fore-and-aft motion stimuli.

**Figure 3:**
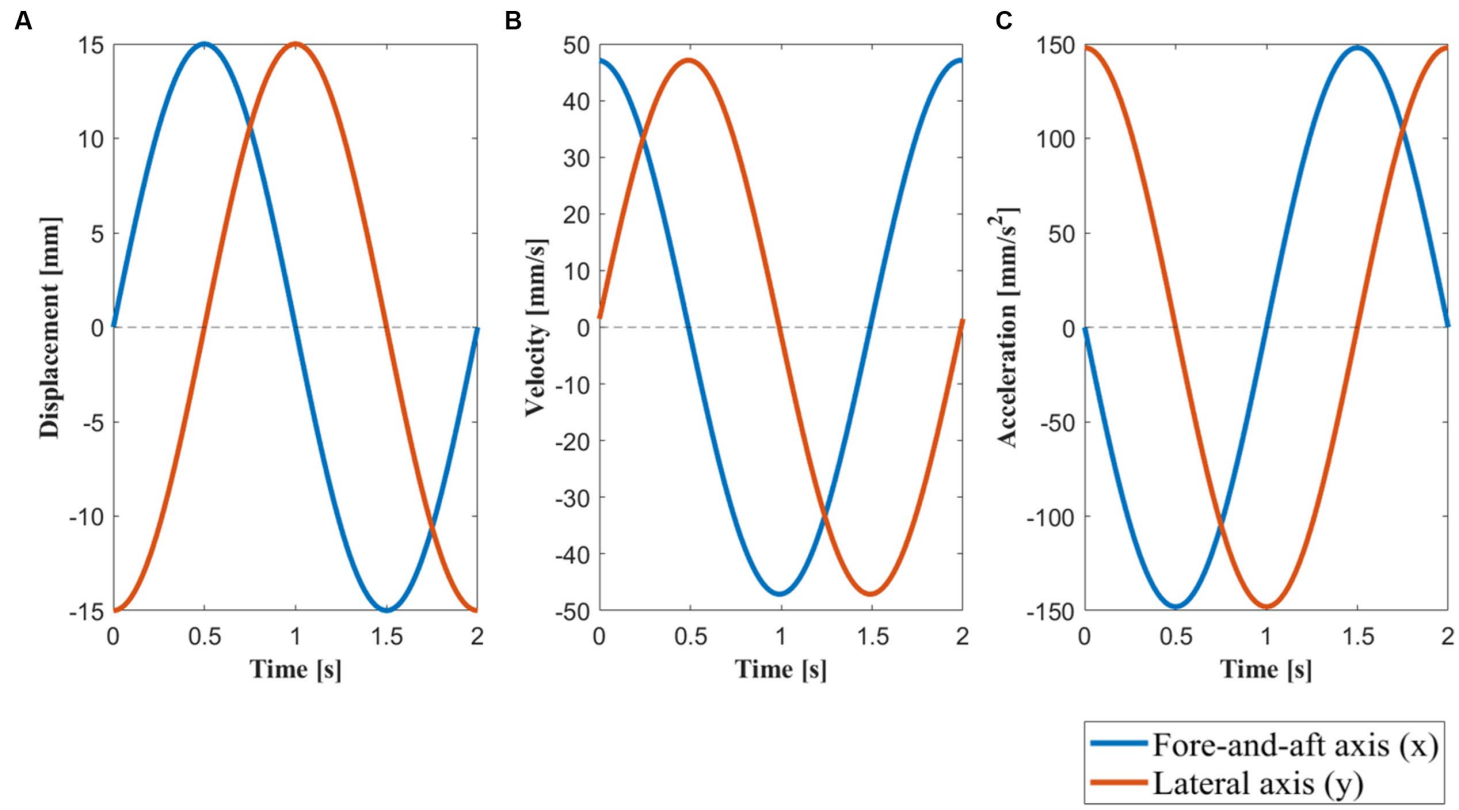
Motion profile along the fore-and-aft axis (x) and lateral axis (y). **(A)** The displacement of the motion platform within one motion cycle with a maximum displacement of 15 mm . **(B)** The velocity of the motion platform within one motion cycle with a peak velocity of 47.12 mm/s . **(C)** The acceleration of the motion platform within one motion cycle with a peak acceleration of 148.04 $\mathrm{mm}/s^{2}$ , which is approximately equivalent to 0.015 g. The blue line represents the motion profile of the motion platform along the forward and backward axis (x) during a 2 s motion cycle, while the orange line represents the motion profile along the lateral axis (y). Under circular motion, there was simultaneous movement in both the x and y directions. Under lateral motion conditions, there was no movement along the y-axis (y = 0), whereas under fore-and-aft motion, there was no movement along the x-axis (x = 0).

In this study, we utilized motion stimuli with a frequency of 0.5 Hz and a peak acceleration of approximately 0.015 g to ensure activation of vestibular responses (36, 37) and to avoid motion sickness-prone frequency ranges (38). As our focus was on the axis of motion, we maintained simplicity in the motion variables. Therefore, we designed three types of motion stimuli, consisting of two one-dimensional linear motion stimuli along the fore-and-aft axis (x) and lateral axis (y), and one circular motion stimulus that combined linear accelerations along the x- and y- axis. In terms of physical principles, the circular motion stimulus is essentially equivalent to off-vertical axis rotation (OVAR) in a steady state (36, 39, 40). The motion stimuli were generated using the motion platform (see Figures 2, 3): forward and backward motion (fore-and-aft), lateral motion (lateral), and circular motion (circular). As a control, a condition with no motion (blank) was included. In summary, four motion direction conditions were tested: circular, lateral, fore-and-aft, and blank.

Lateral motion involved a linear sinusoidal movement at 0.5 Hz perpendicular to the subjects’ facing direction with an amplitude of 15 mm. Fore-and-aft motion involved a linear sinusoidal movement at 0.5 Hz in the direction the subject was facing with an amplitude of 15 mm. Circular motion involved the curvilinear movement along a circular trajectory of a 15 mm radius. Finally, blank motion involved no movement. Detailed motion profiles and parameters of these four conditions are shown in Table 1 and Figure 3. To achieve a gradual change in acceleration between rest and a motion stimulus, the motion patterns were modulated during the first 2 s. Table 1 lists the motion generation functions for the two dimensions (x and y) of the horizontal plane.

**Table 1:** Parameters of the four motion conditions.

Motion conditions	Motion parameters	
	Direction	Motion generation function (period *T* = 2 s)
Circular	Circular movement in the horizontal plane	$x=f\left(t\right)\cos\frac{2\pi }{T}t$ $y=g\left(t\right)\sin\frac{2\pi }{T}t$ $f\left(t\right)=g\left(t\right)=\{\begin{array}{c} \lambda \left(\frac{1}{5}t^{5}-\frac{T}{2}t^{4}+\frac{T^{2}}{3}t^{3}\right),0\leq t\leq T\\ \lambda \left(\frac{T^{5}}{30}\right)=15,\;t>T \end{array}$
Lateral	Left–right motion y=0 in this condition	
Fore-and-aft	For-backward motion x=0 in this condition	
Blank	Not applicable x=y=0 in this condition	

#### 2.3.2 Experiment procedure

This study adopted a block and session design. During each block, a 14 s motion stimulus was presented, followed by a 20 s rest period with no motion. Each motion direction condition consisted of 10 repeats of a block; thus, each condition lasted 6 min. For all moving conditions, subjects were seated inside the motion platform with their head fixed on a chin rest and their feet touching the carpeted floor of the (moving) room. They were instructed to fixate their eyes on a 3 cm × 3 cm red cross presented on a black background that was set at a distance of 1.0 m at the eye level (note that in the enclosed room of the motion platform, the red cross moved in synchronization with the subject’s head). The direction in which the subjects faced did not change for any motion. Each session comprised the four motion conditions that were randomly sequenced. Enough rest time was allowed between successive blocks to avoid fatigue. Each subject participated in three sessions. Sessions were conducted at the same time on different days. NIRS data were collected continuously during the whole session.

#### 2.3.3 Controlled variables

Throughout the experiment, ambient temperature, light, and noise levels were carefully controlled. Room temperature was maintained at 21°C, with the environmental light turned off to keep the room in darkness. All participants wore earplugs to block out external sounds. The physiological status, including heart rate and blood oxygen concentration, of the subjects was monitored using a probe attached to their earlobe.

To isolate the influence of motion artifacts from the cortical signals, we conducted a validation test by placing NIRS probes on a plastic manikin head. The part of the manikin head where the light fibers and detectors were placed was made of a thick silicon gel, and the manikin head was fixed on the headrest inside the motion platform (see Figure 2 and Appendix I.4). During the validation test, the artificial head underwent testing under all motion stimulus conditions, similar to the real participants.

### 2.4 fNIRS data preprocessing and analysis

Prior to data analysis, channel exclusion and artifact rejection were performed. A channel was excluded if the NIRS data failed to exhibit the heart rate pattern (41). Artifact rejection was conducted on a per-channel basis to identify regions with large noise, which could result from shifting of detectors and/or light sources. The contamination in the data was typically caused by a sudden movement of the subject and resulted in a synchronized sudden shift in the light intensity data across different channels. In this study, the light intensity level was analyzed with a two-sided moving standard deviation algorithm to identify noise-contaminated blocks (42). If at least one artifact was detected within a block, the entire block was marked as noise-contaminated and rejected from further analysis.

Following channel exclusion and artifact rejection, the data were processed using HOMER (43). To eliminate high-frequency noise and very slow drift, a third-order Butterworth bandpass filter was applied to the data between 0.001 and 0.3 Hz. In designing the filter, phase distortion was intentionally avoided, as described by Osharina et al. (44). The light intensity level was then converted into the concentration change of oxyhemoglobin (HbO) and deoxyhemoglobin (Hb) through a calculation process based on the modified Beer–Lambert Law (45). A grand average of hemoglobin concentration change was then calculated among the remaining blocks within each motion condition. We extracted the HbO levels for each condition by averaging the concentration change data over a 4 s time window for both the moving period (motion-on) and the resting period (motion-off). As previous research has shown that the HbO levels peak between 5 and 8 s and return to the baseline window of 12 s after stimulus onset (46, 47), we captured the HbO levels during the 6–10 s period following motion onset for the motion-on condition. For the motion-off condition, we selected a 4 s time period during the resting period, which ranged from 12 to 16 s after the movement stopped in each block. Statistical analysis was performed on the averaged HbO levels extracted from the four motion conditions (circular, lateral, fore-and-aft, and blank) with two motion treatments (motion-on and motion-off).

## 3 Results

**Figure 4:**
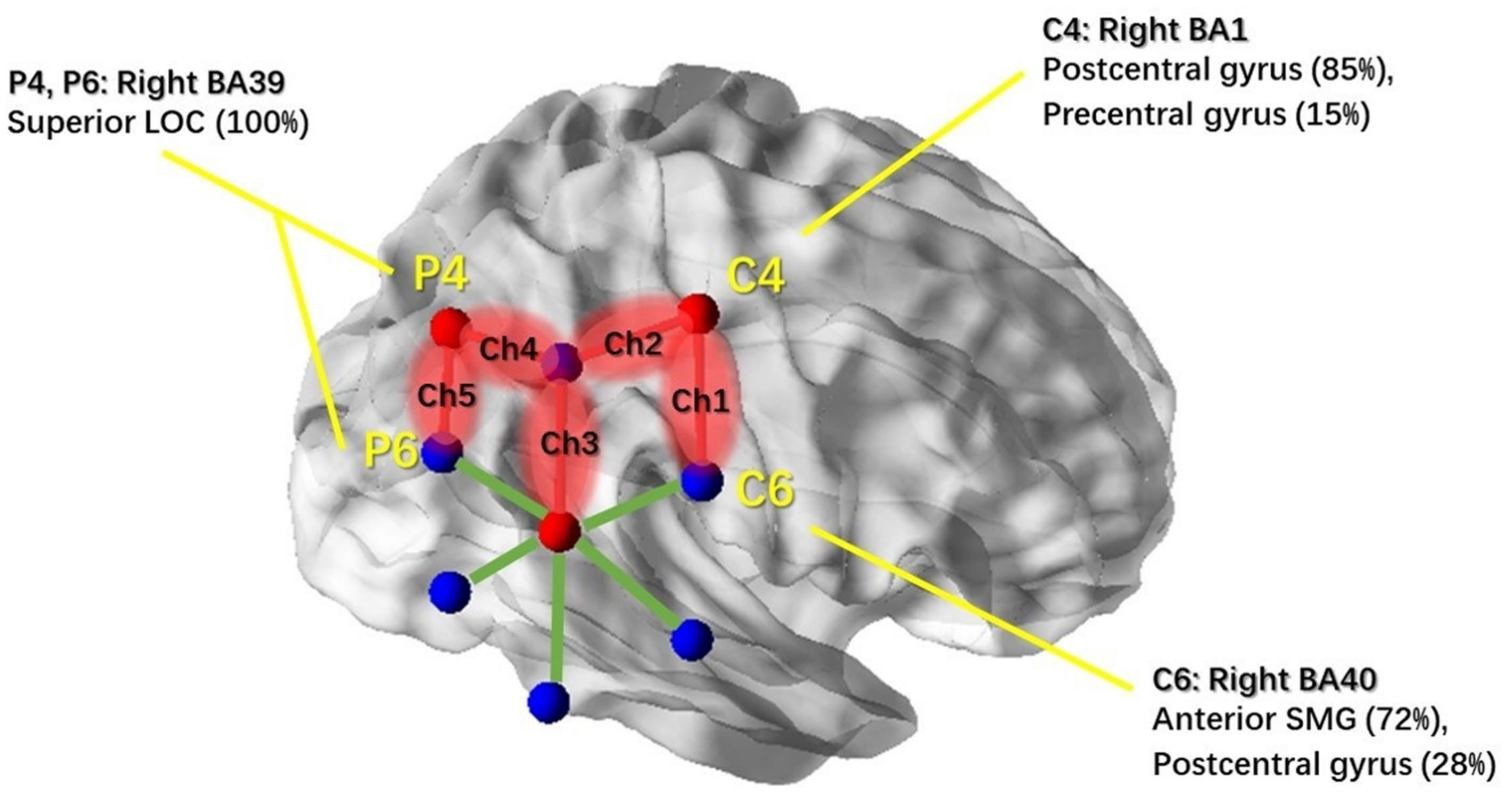
NIRS channels’ response to general motion and associated cortical regions. Red balls indicate the location of the three NIRS detectors. Blue balls indicate the location of the six NIRS light sources. Centers of the red and blue balls were determined by the coordinates of the overlapping 10–20 system markers (C4, C6, P4, and P6); associated brain regions were reported based on the MRI result from Scrivener et al. (2020). NIRS channels that showed significant activation in response to real physical motion stimulation are highlighted in red; non-significant channels are illustrated with green lines. This figure was created using BrainNet Viewer (48).

First, we examined the blank condition. The results revealed that none of the channels were modulated under the blank motion condition during any of the sessions. Then, a three-way repeated measures MANOVA was conducted with three within-subject factors, namely, motion condition (circular, lateral, and fore-and-aft), motion treatment (motion-on and motion-off), and session (repeated measures on three different days) for each channel. We found a significant main effect of “motion treatment” on five NIRS channels (Ch1, Ch2, Ch3, Ch4, and Ch5), with greater HbO levels observed under the motion-on condition compared to the motion-off condition (Table 2 shows statistical results of those significant channels). This suggests that the cortical areas under these channels responded to general motion. Moreover, the linear term of interaction effect on motion treatment (motion-on and motion-off) and motion condition (circular, lateral, and fore-and-aft) was significant for Ch1 [*F*(1,11) =5.958, *p* = 0.033] and Ch4 [*F*(1,11) = 15.018, *p* = 0.003]. Statistical analysis did not reveal any significant main effect or interaction effects with the session, indicating that the data were stable (see Appendix I.2/I.3 for HbO level plots of the session and motion treatment under each motion condition). For visualization, we utilized the BrainNet Viewer (48) to map the brain regions associated with the activated NIRS channels (see Figure 4).

**Table 2:** Summary of statistical results on main effects for motion treatment.

	Statistic parameters				
Channel	df	MS(Effect)	MS(Error)	*F*	Value of *p*
Ch1	(1, 11)	81.921	4.905	4.905	0.049*
Ch2	(1, 11)	171.282	22.105	7.748	0.018*
Ch3	(1, 11)	50.124	10.112	4.957	0.048*
Ch4	(1, 11)	128.024	22.736	5.631	0.037*
Ch5	(1, 11)	70.331	10.526	6.681	0.025*

* < 0.05. MS(Effect) represents the mean square of motion treatment; MS(Error) represents the mean square of error. The scalp locations of all the NIRS channels are shown in Figure 1.

**Figure 5:**
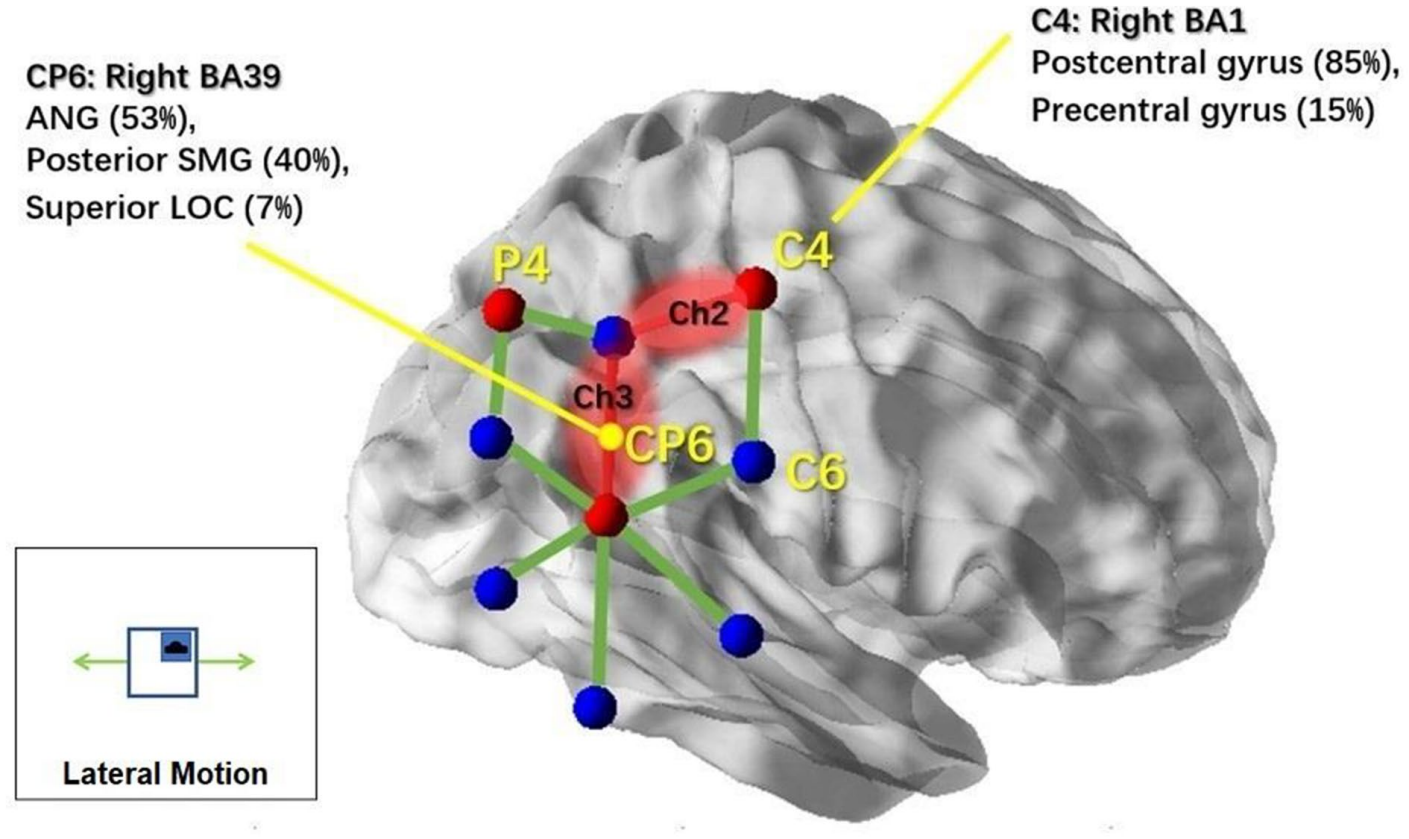
NIRS channels’ response to lateral motion and associated cortical regions. Brain regions associated with the markers (C4 and Cp6) in the 10–20 system were reported based on the MRI result from Scrivener et al. (2020). NIRS channels that showed significant activation in response to lateral motion stimulation are highlighted in red; non-significant channels are illustrated with green lines. This figure was created using BrainNet Viewer (48).

Then, we examined the simple effects of motion treatment within each motion condition. In the lateral condition, we found significant motion treatment effects in channel 2 (Ch2) [*F*(1,11) = 10.081, *p* = 0.002] and channel 3 (Ch3) [*F*(1,11) = 5.267, *p* = 0.025], with increased response observed in the motion-on condition compared to the motion-off condition. To visualize the cortical areas associated with lateral motion, we mapped the activated brain regions under these two channels, as shown in Figure 5, with the assistance of BrainNet Viewer (48).

**Figure 6:**
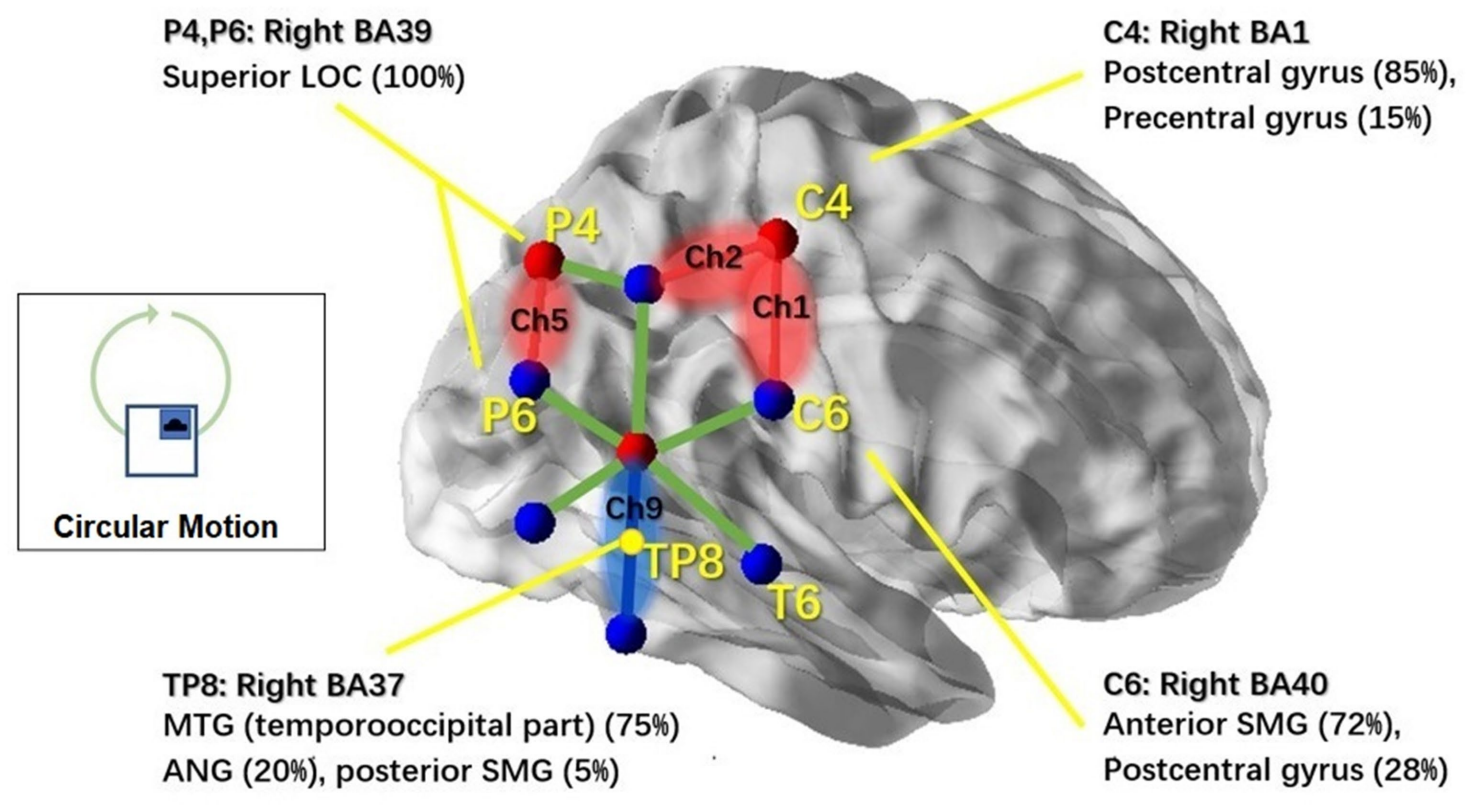
NIRS channels’ response to the circular motion and associated cortical regions. Brain regions associated with the markers (C4, C6, P4, P6, and TP8) in the 10–20 system were reported based on the MRI result from Scrivener et al. (2020). NIRS channels that showed significant activation in response to circular motion stimulation are highlighted in red; Ch6 which showed inhibitive responses is highlighted in blue. Non-significant channels are shown with green lines. This figure was created using BrainNet Viewer (48).

In the circular condition, significant motion treatment effects were observed in channel 1 (Ch1) [*F*(1,11) = 5.511, *p* = 0.022], channel 2 (Ch2) [*F*(1,11) = 4.378, *p* = 0.04], and channel 5 (Ch5) [*F*(1,11) = 4.287, *p* = 0.044]. Channel 4 (Ch4) showed a marginally significant effect [*F*(1,11) = 4.066, *p* = 0.051]. All of these channels showed a significant increase in response to the motion-on condition compared to the motion-off condition. In particular, channel 9 (Ch9) [*F*(1,11) = 6.911, *p* = 0.011] showed a significant decrease in response in the motion-on condition compared to the motion-off condition. To better illustrate the cortical areas associated with circular motion, we mapped the brain regions under those channels, as shown in Figure 6, using the same method as for lateral motion.

No significant channel was found for the fore-and-aft conditions. The control channel was placed on the scalp above the occipital lobe, and the blank control channel did not show any significant results in any of our statistical analyses, indicating the validity of our results. Furthermore, during the validation test, no significant results were obtained from any of the runs using the manikin head.

## 4 Discussion

This study examined the human cerebral cortical hemodynamic response to three different types of passive translational motion stimulation in the horizontal plane: circular motion, lateral motion, and fore-and-aft motion. In this section, we will elaborate on two major findings: (a) the cortical regions responsive to passive translational motion and (b) the different response patterns to the circular motion and the lateral motion. Finally, some limitations and validity considerations are discussed.

### 4.1 Cortical response to general passive motion

Based on the motion effects in the three-way MANOVA test (see Table 2), the cortical region that was responding to general passive motion in the horizontal plane could be characterized by the coverage area under Ch1–Ch5 (see Figure 4). Based on previous studies that established a correlation between positions in the 10–20 system and intracranial structures (49), we identified the areas under channels 1–5 as the Brodmann’s area (BA) 1 (the right postcentral and precentral gyri, both of which are associated with C4); the BA 39 (the right inferior parietal lobe [IPL] which is associated with CP6 and the right superior LOC which is associated with P4/P6); the BA 40 (the right anterior SMG and postcentral gyrus, both of which are associated with C6); and the BA 37 (the temporo-parietal regions which are associated with TP8). These brain areas are highly consistent with the activated brain areas reported in most of the previous studies, even those using different alternative stimuli (15, 16, 19, 21). We acknowledge that subjects were exposed to the whole-body motion during the experiment and that the associated somatosensory stimulation would also contribute to the significantly increased NIRS responses (50, 51).

### 4.2 Differentiated response to different motion conditions

The most interesting finding of this study was that different types of translational motion induced different patterns of response, indicating that different brain regions were activated. In the results, the cortical responses to physical motion were most broadly distributed for circular motion (see Figure 6, including regions under Ch1, Ch2, Ch5, and Ch9). Interestingly, the response in Ch9 corresponds to an EEG study that used physical rotation stimuli (52). The study found that rotation motion evokes a late, long-lasting component with a mean peak latency of 1,800 ms after motion onset. Topographic analysis indicated that this component primarily originates from the bilateral temporo-parietal region, which coincides with the brain area covered by ch9. The resemblance to OVAR of the circular translational stimulus may partially explain this result. The brain’s ability to reconstruct angular motion from the circular movement of the linear acceleration vector, as demonstrated by continuous unidirectional nystagmus during OVAR, suggests that the brain may interpret the stimulus as angular motion despite its translational nature. Further comparative studies between the circular translational stimulus and OVAR could shed light on the neural mechanisms involved in motion perception. For lateral motion, the activated brain regions were more limited and had a smaller range (see Figure 5; mainly regions under Ch2 and Ch3). In the fore-and-aft condition, no significant activated brain area was found. This may be because the cortical regions activated under this condition are located deeper in the cerebral cortex, and fNIRS can only measure the surface cortical hemodynamic activity. In summary, our results suggest that different directions of motion activate different vestibular and somatosensory regions. In previous studies, due to the conflicts between the vestibular system and the other sensory system and, hence, cortical activity that is more widespread than that produced by our natural stimulation, using alternative methods to stimulate the vestibular system may have resulted in the activation.

In terms of hemoglobin level change in the responding channels, that of Ch9 was in an opposite direction compared to other channels. For circular motion, Ch9 demonstrated a decrease in the HbO level, while others showed a general increase in HbO concentrations. These inhibited responses were mainly associated with the TPJ region (see Figure 6), which has also been reported in a previous study (53, 54). Brandt et al. suggested that these responses might be related to the functional inhibitory brain mechanism connecting the visual cortices and vestibular cortices (55). Future studies are needed to confirm the mechanism underlying the inhibited response under the Ch9 region.

### 4.3 Limitations and validations

Although we limited the motion to a diameter, or a maximum point-to-point distance, of 30 mm to reduce the mechanical noise from the motion platform, there is still concern that the hemodynamic data collected may be contaminated by motion artifacts. Nevertheless, the testing conducted with the silicone manikin head revealed that the motion stimuli themselves did not elicit any significant responses in the NIRS system. This suggests that the significant patterns reported in the human experiment are unlikely to be generated by motion artifacts. Although the relative motion between the participants’ head and body was minimized using a headrest and the relative motion between the eyes and head was restricted through fixation, we could not eliminate the possibility of smooth pursuit eye movement and reflexive eye movements (e.g., vestibulo-ocular reflex) of small magnitudes, especially under the lateral motion and circular motion stimulation. Whether such eye movement might contribute to the increases in the HbO level in Ch2/5 will require future experiments.

## 5 Conclusion

According to this study, different specific regions of the brain respond selectively to different types of physical motion. In comparing circular, lateral, and fore-and-aft moving conditions, the circular condition triggered the most widespread brain area, including the postcentral and precentral gyri, superior LOC, and TPJ regions. The brain response to lateral motion was restricted to a smaller range, covering the postcentral and precentral gyri and the posterior SMG. To the best of our knowledge, this is the first study to demonstrate the selectivity of human brain regions in response to different directions of motion. The responding brain regions are consistent with the areas that were previously shown to be activated in the cerebral cortex using other vestibular-stimulating methods in previous studies.

## Data availability statement

The raw data supporting the conclusions of this article will be made available by the authors, without undue reservation.

## Ethics statement

The studies involving humans were approved by Hong Kong University of Science and Technology. The studies were conducted in accordance with the local legislation and institutional requirements. The participants provided their written informed consent to participate in this study.

## Author contributions

YZ: Conceptualization, Data curation, Formal analysis, Investigation, Methodology, Project administration, Software, Validation, Writing – original draft, Writing – review & editing. YuW: Conceptualization, Data curation, Formal analysis, Funding acquisition, Investigation, Methodology, Software, Validation, Visualization, Writing – original draft, Writing – review & editing. YiW: Investigation, Validation, Visualization, Writing – review & editing. RS: Conceptualization, Funding acquisition, Supervision, Validation, Visualization, Writing – review & editing. CC: Conceptualization, Resources, Writing – review & editing. RC: Conceptualization, Resources, Writing – review & editing. AW: Conceptualization, Writing – review & editing.
