# Supplementary material for: Identification of the human cerebral cortical hemodynamic response to passive whole-body movements using near-infrared spectroscopy
Source: Front Neurol. 2023 Dec 13;14:1280015. doi: 10.3389/fneur.2023.1280015 (PMC10751349; doi:10.3389/fneur.2023.1280015)
Supplement: Supplementary file 1 [file Image_1.PDF]

Appendix I

Appendix I.1 Illustration for channels activated by general motion.

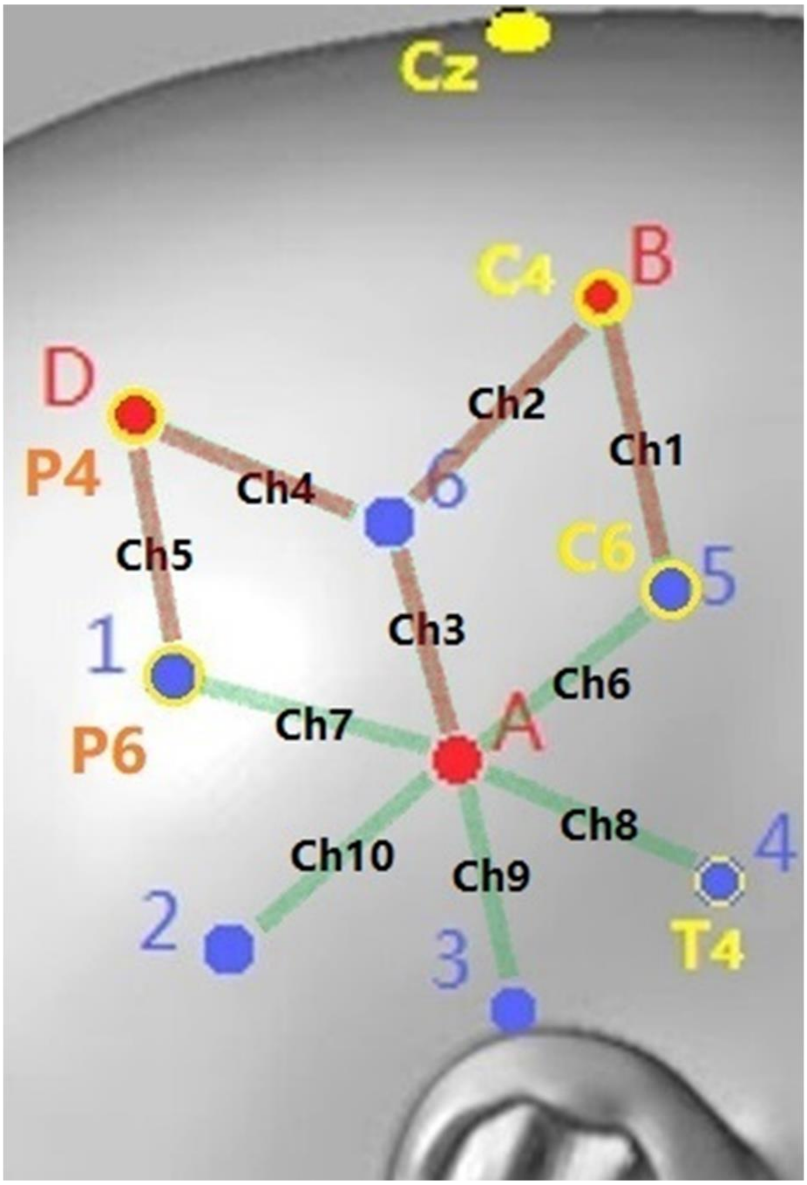

Appendix I.2 Motion treatment plotted against sessions for all channels activated by lateral motion.

Channel 2

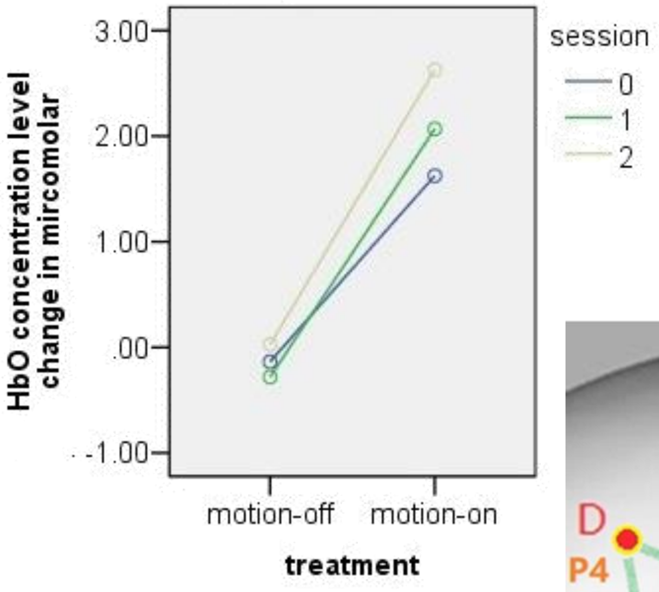

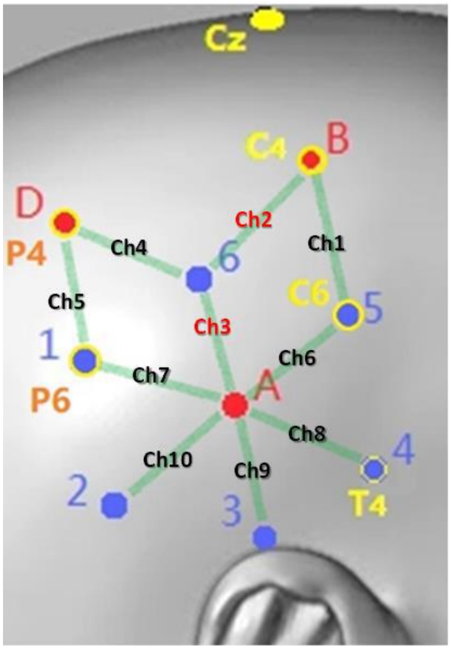

Channel 3

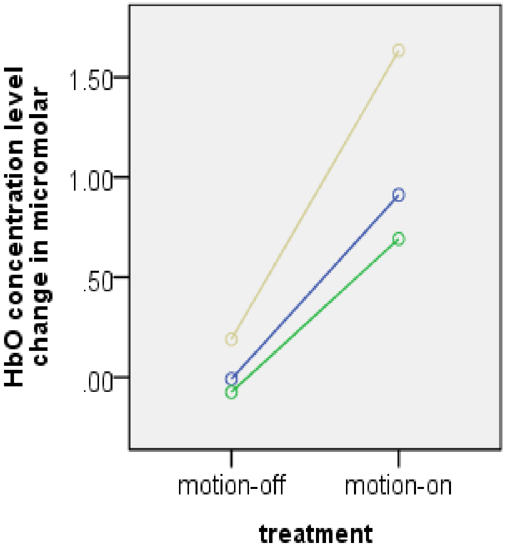

Appendix I.3 Motion treatment plotted against sessions for all channels activated or inhibited by circular motion.

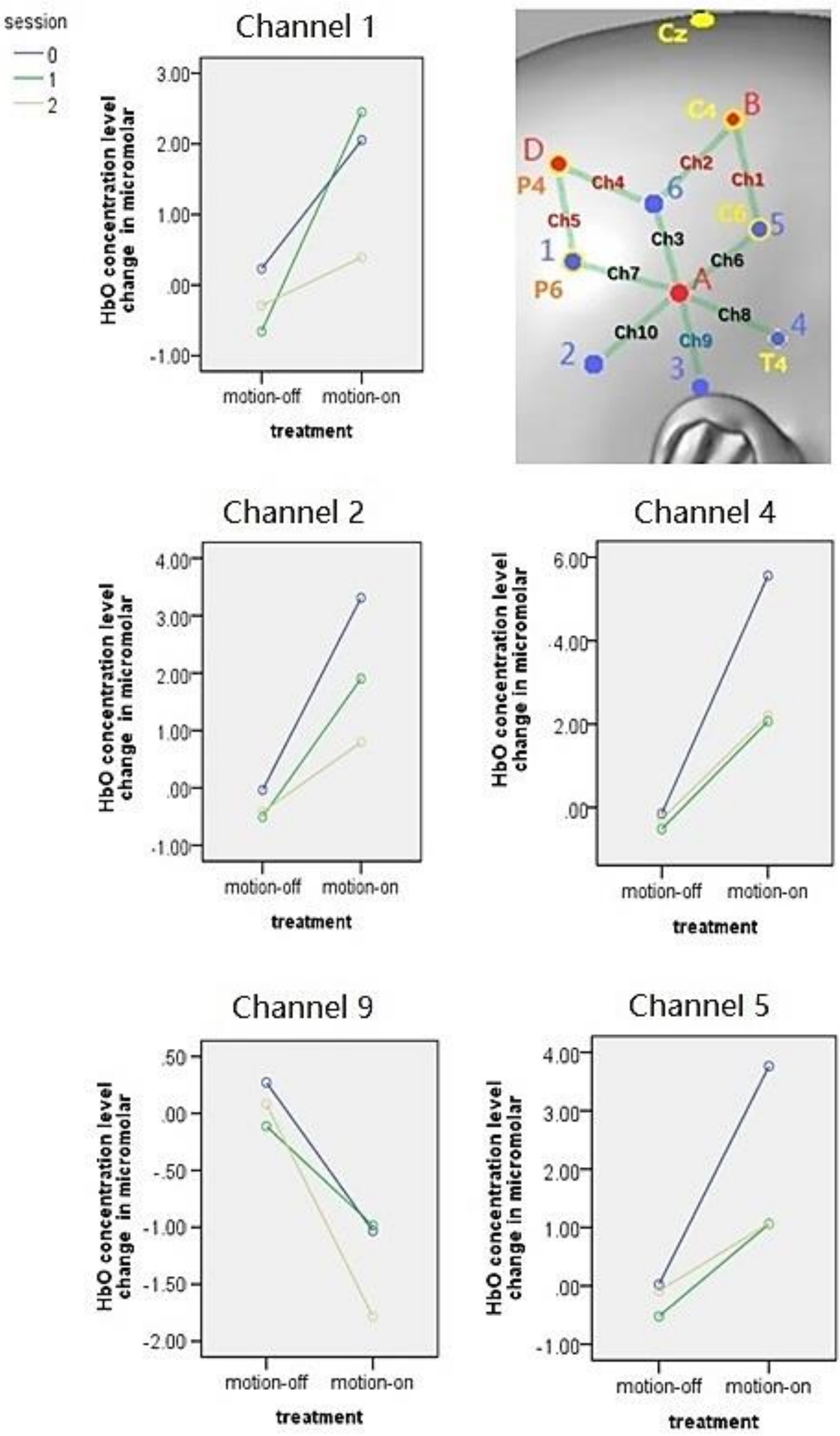

Appendix I.4 Manikin head in the motion platform for validation test

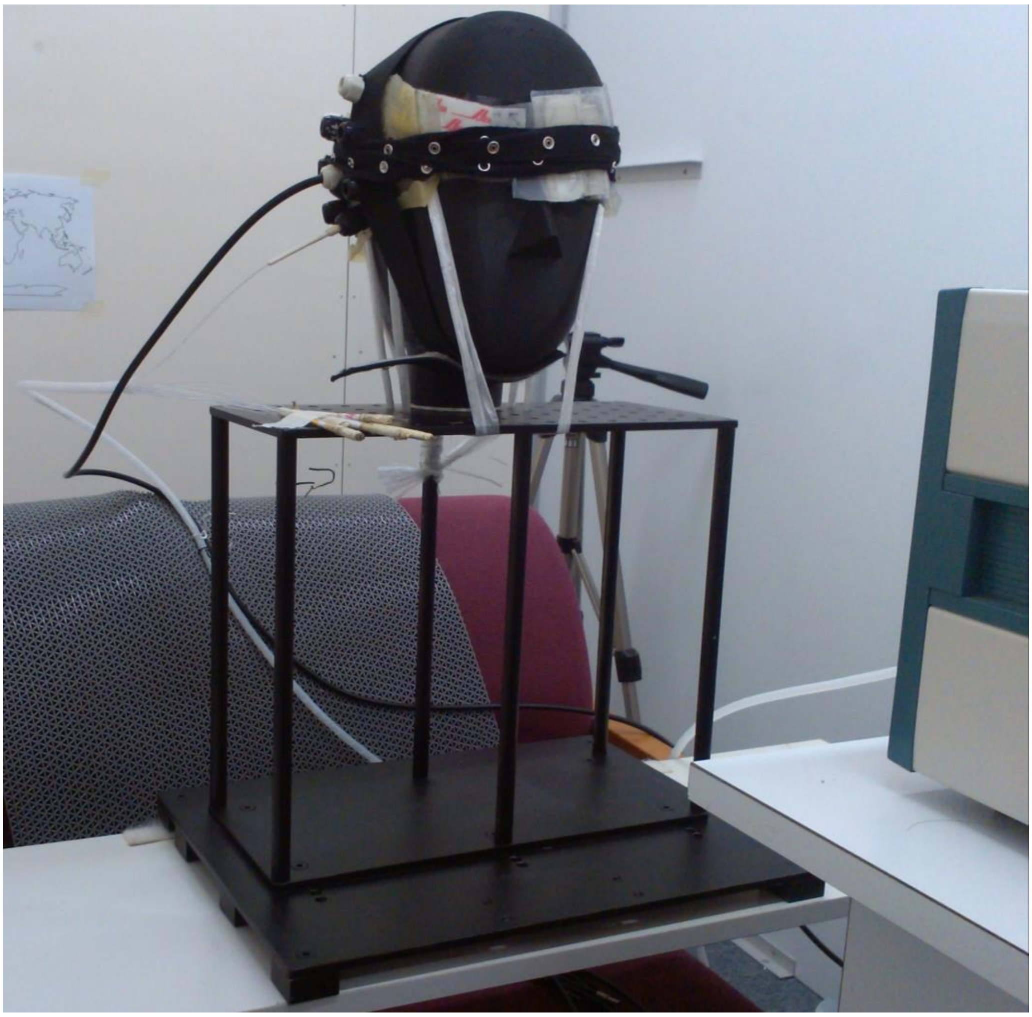

Appendix I.5 Motion platform

The motion platform locates at CLP Power Wind/Water Tunnel Facility in the Hong Kong University of Science and Technology. A 4m x 3m fully enclosed test platform was supported on two pairs of custom-built sliding bearings riding on the precision machined and leveled rails. It could move ±800 mm along the long axis and ±400 mm along the short axis. The motion could be bi-directional narrow band random motions. The maximum acceleration could be generated is around 30 milli-g and the frequency range is 0.05 to 1 Hz. Following is a photo of the motion simulator used in the study. The motion simulator could move along the fore-and-aft axis (x) and lateral axis (y). It is designed and built in house at HKUST. The actuation is electromagnetic.

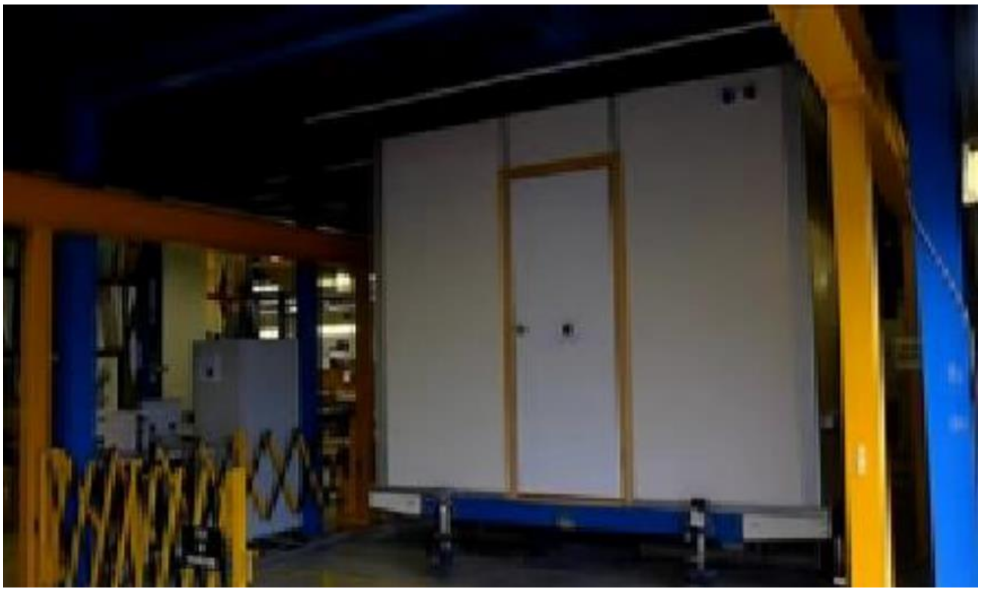

The motion in this experiment is achieved by inputting the coordination file for both axis. The motion parameters must be set within the range of motion simulator capability. Smoothing function should be applied to avoid discrepancy in acceleration 124 especially during the starting and ending period of motion because jerks could be sensed if acceleration is not continuous.
